# The Underlying Social Dynamics of Paradigm Shifts

**DOI:** 10.1371/journal.pone.0138172

**Published:** 2015-09-29

**Authors:** Carlos Rodriguez-Sickert, Diego Cosmelli, Francisco Claro, Miguel Angel Fuentes

**Affiliations:** 1 Facultad de Gobierno, Centro de Investigación en Complejidad Social, UDD, Santiago, Chile; 2 Escuela de Psicología, Facultad de Ciencias Sociales, Pontificia Universidad Católica, Santiago, Chile; 3 Centro Interdisciplinario de Neurociencias, Pontificia Universidad Católica, Santiago, Chile; 4 Facultad de Física, Pontificia Universidad Católica, Santiago, Chile; 5 Santa Fe Institute, 1399 Hyde Park Road, Santa Fe, New Mexico 87501, United States of America; 6 Instituto de Investigaciones Filosóficas, Buenos Aires 1428, Argentina; 7 Universidad San Sebastián, Santiago 7510157, Chile; Université Toulouse 1 Capitole, FRANCE

## Abstract

We develop here a multi-agent model of the creation of knowledge (scientific progress or technological evolution) within a community of researchers devoted to such endeavors. In the proposed model, agents learn in a physical-technological landscape, and weight is attached to both individual search and social influence. We find that the combination of these two forces together with random experimentation can account for both i) marginal change, that is, periods of normal science or refinements on the performance of a given technology (and in which the community stays in the neighborhood of the current paradigm); and ii) radical change, which takes the form of scientific paradigm shifts (or discontinuities in the structure of performance of a technology) that is observed as a swift migration of the knowledge community towards the new and superior paradigm. The efficiency of the search process is heavily dependent on the weight that agents posit on social influence. The occurrence of a paradigm shift becomes more likely when each member of the community attaches a small but positive weight to the experience of his/her peers. For this parameter region, nevertheless, a conservative force is exerted by the representatives of the current paradigm. However, social influence is not strong enough to seriously hamper individual discovery, and can act so as to empower successful individual pioneers who have conquered the new and superior paradigm.

## Introduction

The dynamics of scientific progress and technological evolution involve a sequence of periods of stagnation (deadlocks) and marginal, incremental change followed by brief periods of radical increase in performance accompanied by a swift adoption of a new and superior paradigm. These radical transitions or paradigm shifts involve both a change in the activity of the members of the scientific community and in knowledge itself [1–4].

In order to investigate the underlying mechanics of the non-linear dynamics that characterize the evolution of knowledge, one can linger either on the structure of the technology space itself or, alternatively, focus on the structure of the learning and discovery process of the community of researchers involved in the creation of knowledge. Within the former framework, Silverberg and Verspagen [5] analytically show that the accumulation of complementary inventions can explain radical change in the evolution of technology. Within the latter framework, Kuhn argues that, once indoctrinated into a paradigm, scientists devote themselves to “mopping up” this paradigm by solving puzzles whose solutions reinforce and extend the scope of the paradigm. During this period, anomalies and phenomena for which the paradigm cannot account–or that directly contradict it–are often ignored. However, as these anomalies accumulate, it becomes more likely that a genial maverick will posit an alternative paradigm that will prevail over the old paradigm, in spite of the resistance that the “moppers” of the old paradigm might exercise. Lakatos and Feyerabend, influenced by Kuhn, also considered futile any epistemological exercise that neglected the underlying scientific communities. Yet they differed on their understanding of the scientific activity and its achievements. Lakatos [6] proposed that Science organizes itself in “research programs.” The community of scientists that participate in a particular program defend its hard-core hypothesis from the appearance of anomalies by continuously revising its auxiliary hypothesis. As long as this revision process leads to novel predictions, the paradigm is “progressive.” If only ad hoc explanations are achieved, the program is “regressive.” Maverick scientists therefore start to search for a new progressive program to replace it. To the extent that evidence about the superiority of the new program accumulates, the community of followers of the new paradigm grows at the expense of the old paradigm. Eventually, the old paradigm is abandoned and the vast majority of scientists adhere to the new one. Feyerabend [7] also considered that science was mainly a collective endeavor. However, he denied the existence of a scientific method, and consider that it was impossible to judge the quality of scientific theories by appealing to empirical evidence. Instead, Feyerabend claimed that the supporters of different paradigms used unstructured rethoric where anything goes. He compared the replacement of one paradigm by another to the replacement of an older myth by a newer myth.

In our attempt to contribute to the understanding of the evolution of knowledge, we focus on the structure of the learning process of the scientific community, while emphasizing the social character of this process [8, 9]. Our aim is to account for radical change without resorting to individual grandeur, but rather to the complementarities of individual adventurers. Thus, we aim to account for the paradigm shift involved in the transition, for example, from a Ptolemaic cosmology to a Copernican cosmology without giving centrality to Copernicus in our explanation. In other words, we approach the creation of knowledge mainly as a social enterprise.

Our model is consistent with the Agent-Based modeling approach advocated by Smith and Conrey to explore social phenomena [10, 11] and is informed by two bodies of literature: i) models of technological evolution as a search process in an exogenous physical technological landscape [12, 13]; and ii) models of adaptation to the environment in which both individual search and social influence are used by the agent to choose among alternative technologies [14–17]. Our framework presents the scientific enterprise in a way that is closer to Lakatos’ view. However, similar social dynamics in our world are the result of the social interaction of far less sophisticated agents.

We extend Kauffman et al.’s model [12] in which agents use a hill-climbing algorithm and occasionally randomly experiment in order to consider the possibility that the movement of each individual along the landscape is also informed by the experience of other members of its community. We specifically consider a homogenous community of knowledge exploring a physical landscape via: (i) individual search, (ii) social influence and (iii) experimentation; and study how the relative weight that agents place on the experience of their peers (social influence) with respect to their own experience (individual search) affects the characteristic time of the migration of the members of the community of knowledge from the current paradigm to a new and superior paradigm, i.e., a paradigm shift.

## Analysis and Results

In our model, a homogenous knowledge community explores an exogenous unidimensional continuous physical technological landscape (PT landscape) in which any location *x* represents a specific technology or theory and the predictive power/performance of the theory/technology. The creation of knowledge is modeled as a search process of the members of the knowledge community on this PT landscape *V*(*x*).

### The physical-technology landscape

**Fig 1:**
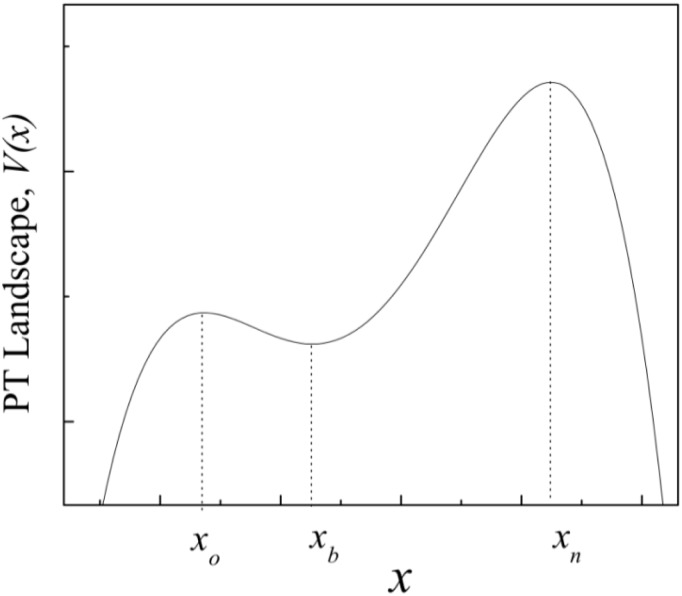
Physical technology landscape, *V*(*x*), in which the agents of the community of knowledge learn. Each point *x* represents a particular theory/technology. Points to the left of the valley minimum *x*_*b*_ represent theories/technologies which belong to the old paradigm, which achieves maximum value at the local optimum *x*_0_, and points to the right represent theories/technologies which belong to the new paradigm, which achieves maximum value at the global optimum *x*_1_. Initial conditions involve *n* agents at the current paradigm *x*_0_.

The PT landscape has a local optimum on *x*_0_ (current paradigm) and a global maximum on *x*_*n*_ (new paradigm). Then, each hill represents a scientific (technological) paradigm see Fig 1.

As an illustration of the underlying idea of the PT landscape, consider a simplified account of high jump techniques, where one can characterize each technique by one single parameter, namely, the angle of the athlete’s head when jumping towards the bar. Before the Olympic Games held in Mexico City in October 1968, the straddle technique dominated athletic competitions, but one innovator, Dick Fosbury, revolutionized the discipline when he used the now-called Fosbury flop technique to win the Olympic gold medal. After this event, the technique began to spread around the world in a continuous fashion, and soon, floppers were dominating international high jump competitions due to the advantages provided by this technique. Unlike the straddle technique, where athletes face down as they roll over the bar, the floppers face upwards. In terms of our model, a straddler jumps towards the bar at a 45° angle, and a flopper jumps at a 135° angle. In Fig 1, those values would represent the local and global maximum, respectively.

To model the PT landscape, we have used a quartic polynomial form that takes into account the above-mentioned features of the proposed topology:

$$\begin{array}{c} V(x)=a(-x(x+b)(x-c)(x-d)+e) \end{array}$$ (1)

With *a* = 10^−2^, *b* = 2, *c* = 1, *d* = 6 and *e* = 2 × 10^2^. This potential gives

$$\begin{array}{c} f\left(x\right)=V^{\prime }\left(x\right)=-\gamma \left(x-x_{n}\right)\left(x-x_{b}\right)\left(x-x_{o}\right) \end{array}$$ (2)

Where *γ* = 0.04 and *x*_*o*_ ≈ −1.27, *x*_*n*_ ≈ 4.5, *x*_*b*_ ≈ 0.5 are the values of the position of the old and new paradigm, and the bottom of the valley respectively. Following Kauffman [12], we have adopted here a set of parameters that produce a PT landscape, *V*(*x*), with a local and a global maximum, i.e. and inverted double well potential.

### Exploration of the PT landscape by the knowledge community

Movement along the landscape is governed by individual search combined with social influence: each agent weighs its own experience against the experience of the other members of the community. For the initial conditions, we assume that all members of the knowledge community are working under the current paradigm in the vicinity of *x*_0_. One can interpret this location as the result of path dependency (when, for instance, the previous generation and their mentors were searching in this area). Experimentation in all cases takes the form of white noise. Specifically, we modeled the evolution equation on the PT landscape for the *i* ^*th*^ agent by

$$\begin{array}{c} \frac{dx_{i}}{dt}=\left(1-\alpha \right)f\left(x_{i}\right)+\alpha \omega g\left(x_{i},x_{j}\right)+\sqrt{\varepsilon }\;\xi \left(t\right) \end{array}$$ (3)

Where *x*_*i*_ with *i* ∈ 1, …, *n* is the position of agent *i* at the instant *t* navigating the PT landscape *V*(*x*);

1. *f*(*x*_*i*_) = *V*′(*x*_*i*_), represents individual search;
2. $g(x_{i},x_{j})=\frac{1}{m}\sum_{j=1,j\neq i}^{m}\left[V(x_{j})-V(x_{i})\right](x_{j}-x_{i})$, represents social influence

Where *x*_*j*_ with *j* ∈ {1, …, *m* ≤ *n* − 1} represents the position of an agent *j* randomly sampled by agent *i*; *α* gives the weight that agents attach to social influence; *ω* is the parameter that takes into account the strength differences between the influence of information obtained from the experience of the researcher’s peers and information obtained from his own inspection of the technological landscape; *ξ*(*t*) represents the way in which agents experiment; in this case, a zero means Gaussian white noise with strength *ɛ*.

Individual search is described here by a standard hill-climbing algorithm. Implicitly, we assume that agents have only local knowledge of the structure of the landscape, and non-local information can be gathered only through the performance of other members of the community. Each sampling involves an individual observing the location of her fellow searcher and provides an accurate estimation of the value function at this location. For an observed Δ*V* = *V*(*x*_*j*_) − *V*(*x*_*i*_), he gets closer to the sampled agent if it is a successful one and moves away otherwise. The final effect of social influence will be the result of the aggregation of the reactions against each sample.

In order to understand the logic of the search process, imagine a group of mountaineers trying to reach the highest altitude in a chain of mountains whose topology is unknown to them and visibility is limited due to weather conditions. Individual search in our model works as though each mountaineer could only observe the direction (right or left, in our one-dimensional landscape) towards which the mountain on which she is located gains altitude. If this was the only force influencing the path chosen by the mountaineer, those located towards the left of *x*_*b*_ would end up at {*x*_0_, *V*(*x*_*o*_)} and those located towards the right of this point would end at {*x*_*n*_, *V*(*x*_*n*_)}. From time to time, the mountaineer will make a random step. If such a step (or set of steps) leads her to trespass the valley, the agent can end up on a summit that did not correspond to the hill on which she was located. Under the same metaphor, a socially influenced search can be understood in the following way: although clouds prevent mountaineers from observing how their fellows are doing, they can still use their voices to ascertain their peers’ location by shouting, “Is there anybody out there?”. When a fellow mountaineer responds to this call, she can infer its location and altitude. If the answer comes from a higher altitude, the mountaineer will try to get closer to the source; otherwise, she will move away. Indeed, how much our mountaineer believes in her peers will critically determine the interaction of these different forces.

### Paradigm shifts

The main output of the simulation is the average amount of time that passes before the whole community moves from *x*_0_ to *x*_*n*_, for different values of the social learning parameter *α* and for different population sizes *n*. By construction, we rule out the possibility that an agent migrates from the new paradigm back to the old paradigm. If this assumption was relaxed, the system would converge to stationary distribution in which agents are most of the time at the new paradigm. In order to compute the time for this paradigm shift, we compute the average time, *t*_*s*_, that it takes for the complete community to go from *x*_0_ to *x*_*n*_. We varied the value of *α* from 0 to 1, and used three different population sizes: 12, 24 and 36 and *m* = 11, 23 and 35 respectively. The rest of the parameters were fixed to, *ɛ* = 0.37, and *ω* = 3.14. We have chosen a set of parameters that will give similar times to go from one optimum to the other in an extreme situation, i.e. *α* = 0 and *α* = 1. We performed 200 simulations for each parameter combination (see S1 File)

In the simulations, the difference in altitude between both peaks of the PT Landscape represent the gains in predictive power or technological performance when moving from the old to the new paradigm. The valley, on the other hand, could be understood as the costs of the transition than an agents faces when during the migration to the new paradigm. Changing the parameters of the quartic polynomial in such a way that either increases the difference between the local and the global optima or deepens the valley between them will increase the characteristic time for a paradigm shift to occur. Qualitative features of the model, regarding the comparative statics of social influence are robust to these changes.

**Fig 2:**
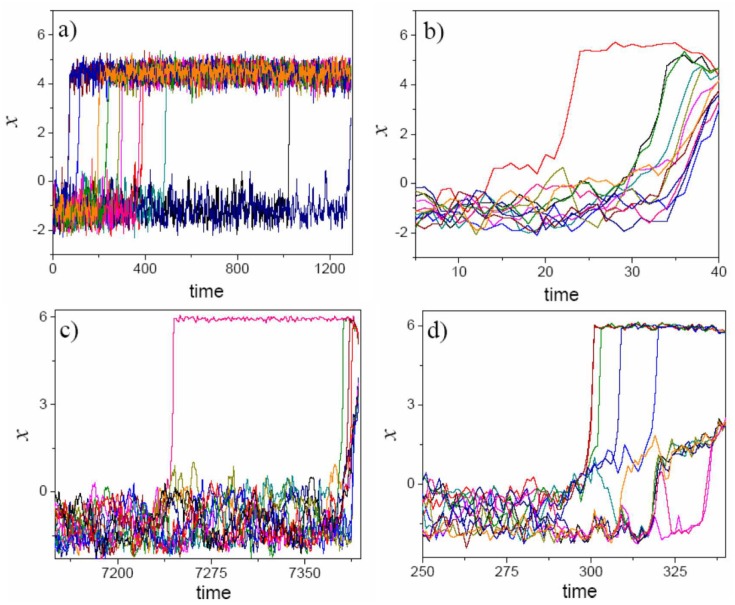
One realization of Eq (1) for different values of *α*. **(a):** *α* = 0, **(b):** *α* = 0.15, **(c):** *α* = 0.7, **(d):** *α* = 1.

**Fig 3:**
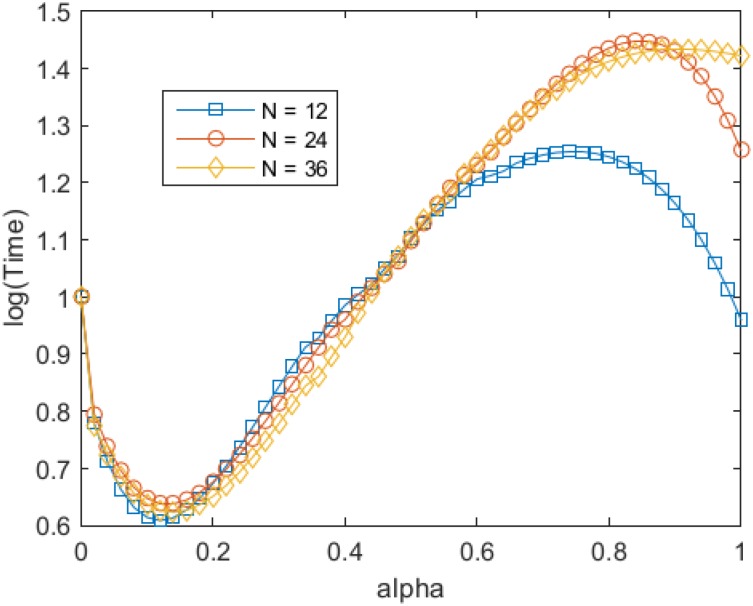
Mean time to paradigm shift, as a function of *α* (the social learning parameter), for different values of *n* (population size). Three different regimes can be observed for all values of *n*. For low values of *α*, which means that most learning is individual, an increase in *α* reduces the mean time to paradigm shift, until a minimum is achieved. For intermediate values of *α*, an increase in *α* increases the mean time to paradigm shift, until a maximum is achieved. Finally, for high values of *α*, which means that most learning is social, an increase in *α* decreases the mean time to paradigm shift.

Representative simulations of the transition from the old paradigm to the new paradigm are presented in Fig 2. The average characteristic times for different values of the weight that agents attach to different values are presented in Fig 3. The MATLAB code available is provided as supporting information that. It allows alpha to be changed continuously from 0 to 1, and the final output is the trajectory of each agent, following the proposed dynamics.

In the absence of noise, *ɛ* = 0, it is clear that there are trivial dynamics in the community of agents, and the whole community will be concentrated at *x*_0_ (the initial condition is set at the neighborhood of this point). However, if noise is introduced in the form of either experimentation or random mistakes, the asymptotic distribution of the community’s location will involve the whole community being at *x*_*n*_, irrespective of the weight that agents posit on social influence. When agents do not place any weight on social influence, all that is required is a set of random steps that allows each individual to cross the valley. The rest of the job is done by the hill-climbing strategy, whether facilitated or not by the social structure. Notice, however, that individual migrations in the absence of social influence are independent from each other, so non-linear shifts are not observed (see Fig 2a).

The introduction of social influence produces two effects. On the one hand, it increases the conservative attraction force that researchers working under the old paradigm exert on possible innovators, extending the time before the transition towards the new paradigm begins. On the other hand, it increases the revolutionary attraction force that pioneers who conquer the superior paradigm exert on the mass of researchers still working under the old paradigm. The more pioneers that conquer the new summit, the stronger this effect becomes. Accordingly, the non-linear character of the migration process is observed in Fig 2b and 2c, which is consistent with the phenomenology of the paradigm shifts previously discussed. The mass of agents concentrated on the inferior paradigm forces social influence to work together with individual search, thus preventing migration towards the alternative and superior paradigm. When a pioneer manages to cross the valley, individual search will now help him to climb to the new paradigm. Until the pioneer reaches an altitude greater than the local optimum at *x*_0_, social influence works against the individual’s hill-climbing algorithm. When the pioneer overcomes this threshold, social influence starts working in the same direction as individual search. As successful pioneers make it to the global maximum, they become an attraction force towards the new paradigm. Notably, once a critical mass of pioneers has migrated to the new paradigm, social influence stops being a conservative force on the system as a whole, thus triggering a swift and accelerated migration from the rest of the community towards the new paradigm (follow-the-leader effect).

The emergence of the three regimes described in Fig 3 highlights the non-trivial interaction of the conservative and revolutionary forces that underlie the movement of the members of the knowledge community along the PT landscape. When knowledge dynamics are driven only by individual experience and experimentation, migration towards the new paradigm is linear (see Fig 2a). The absence of social influence makes it easier for the first pioneers to abandon the old paradigm, but these pioneers exert no influence on researchers who keep working under the old paradigm: each researcher, with independence from the experience of his/her peers, will have to discover the new paradigm on his/her own. We consider this regime ((a) in Fig 3) as the baseline scenario. Non-linear dynamics are observed only for positive values on the weight that agents attach to the experience of their peers (*α* ≠ 0). Now, and as shown in Fig 2b and 2c, a period of marginal change (few explorers discover the superior paradigm) is followed by a brief period of increase in discovery accompanied by a swift migration to the new and superior paradigm when enough individual explorers have made it across the valley.

Fig 3 summarizes the behavior of the model when varying the weight of social influence in terms of the characteristic (average) time for the occurrence of a paradigm shift. With the appearance of even little social influence a steep drop in characteristic time is observed ((b) Fig 3), thus facilitating discovery of the new paradigm by the community. As the weight that explorers assign to their partners increases, average time will begin to increase again, remaining below baseline scenario until *α* ≈ 0.4. Beyond this value of *α*, social influence becomes detrimental and the average time to discover the new paradigm increases because the system behaves more conservatively. This increase in characteristic time persists until *α* reaches values around 0.7 which produces the most conservative behavior of the community. If social influence continues to increase, a third regime takes hold and an interesting phenomenon occurs: a progressive return to baseline behavior as collective migration begins to appear and the community ends up behaving as a single agent when *α* = 1.

Finally, when comparing for different community sizes (*n* = 12, 24 and 36), although the three regimes identified for the smaller community persists for all values of *n*, an interesting result emerges as group size increases. Whereas the characteristic time for lower levels of alpha does not significantly change, paradigm shifts are less likely to occur for higher levels of alpha as the size of the community increases. Greater communities, we suggest, cannot migrate as efficiently as smaller communities when their members are highly coupled because, on the one hand, the social influence felt by mavericks (which are necessary to drive migration) becomes much more conservative (i.e. less variable) and, on the other, because mavericks are less capable of pulling a comparatively “heavier” load.

## Discussion

We have presented a model of scientific progress and technological evolution based on a collection of unremarkable agents that experiment and are subject to varying degrees of social influence. In appealing to the idea of a collection of simple agents fueling scientific progress (in contrast to lone geniuses), it is important to note that we are not advocating a purely social explanation of scientific or technological discovery. Indeed, our model still depends on at least one agent being able to discover a better solution and making such discovery available to its peers. Our main point is not that scientific discoveries *do* happen through the random exploration of ungifted–but socially connected–individuals, but rather that they *could*.

We have therefore chosen to simplify the nature of the individual’s processes in order to highlight what can be produced by social influence. Accordingly, we make no claims on the psychological processes of the explorer who leads the way of discovery, except from the minimal capacity to distinguish the slope of the local landscape (i.e. the usefulness of the technology, the explanatory power of the theory, etc.) In this sense, our model would appear to stand in contrast to cognitive approaches, such as Thagard’s conceptual change model, which builds on a formal description of what is going on in the explorer’s head throughout the discovery process [18]. Specifically, Thagard’s approach highlights the computational mechanisms that might explain how the individual researcher undergoes a conceptual reorganization that enables her to produce a new theory with higher explanatory coherence than its predecessors. However, we do not believe that these approaches are incompatible. While the psychological/computational approach might explain how one individual undertakes his or hers exploratory and individual search process, for a true paradigm shift or technological evolution to obtain, the community of researchers must “come along” and adopt the new epistemological or technical framework. Thagard himself discusses at least two phases of scientific revolution: replacement by discovery (which is mainly an individual process) and development and replacement by instruction (which only happens if others accept the new paradigm, i.e. social influence). Thagard’s approach is of course particularly well suited given the examples he aims at explaining (Copernicus’ heliocentric model, Newtonian mechanics, Lavoisier’s oxygen theory, Darwin’s theory of evolution, etc.) But consider less transcendent instances of technological evolution such as the development of the mountain bike discussed by Sawyer [19]. Here, a series of modifications introduced by individuals that had access to each other’s contributions, ended up producing one of the most notorious changes in the way bicycles are constructed and used (*Ibid*., p5). No one individual undertook the entire process of discovery and then communicated it to its peers, but rather the technological innovation came about as a consequence of individual (local) exploration and distributed collective interaction.

In a similar vein to the previous considerations, an important aspect of our model to keep in mind is that individual agents have no memory of their actions. This is of course not the case for healthy human researchers. A natural and–in principle–tractable extension of the current model would be to endow agents with some capacity to learn from their previous experience, both their own and that of the community [20]. It would be interesting to evaluate if and how such addition might change the dynamics of the exploration and technological progress. Nevertheless, it is notable that even such unremarkable agents, when the interplay of local vs social influences is adequate, can behave as a successful research community.

A counterpart to these cognitive considerations is the eventual utility of a model such as the one presented here to capture the discovery dynamics of a single person while considering that her brain behaves as a collection of agents (i.e. neurons). Indeed, similar approaches based on Potential Landscapes (but not Multi-agent) have been used to model cortical coordination dynamics in a diversity of motor and perceptual situations [21, 22]. It is worth noting that insight-type problems exhibit a similar dynamics to that of paradigm shifts discussed here: a steady but slow increase of comprehension, eventual stagnation when the deductive solution cannot be found and a sudden, non-linear shift to a new way of seeing the problem and the corresponding solution [23].

It is important to consider that we are working with two explicit assumptions regarding the nature of the knowledge landscape that agents can discover. First, we consider that the landscape is fixed, and second, that it is continuous between alternative paradigms. From an epistemological perspective, the latter assumption is a departure from the ideas of Kuhn who believed that alternative paradigms were incommensurable and thus, no *V*(*x*) could be agreed upon among scientists working under different paradigms. In this sense, our model is consistent with the view that the empirical evidence should allow different scientists to agree on the predictive value of a particular theory [24, 25]. Indeed, the amount of empirical evidence will determine the resolution of the landscape [26]. On the other hand, the former assumption implies that our model cannot take into account sociocultural effects on what is considered a successful technology or explanatory paradigm itself [27].

Newton once said: “If I have seen further, it is by standing on the shoulders of giants.” For Newton, it is the unidirectional influence that exceptional geniuses exert on the rest of the members of the community that underlies paradigm shifts. By contrast, our results suggest that mutually informed individual adventurers within a community of far-from-genius researchers can replace the role of superior minds in discovering new and superior paradigms. Richerson and Boyd [28] claim, rephrasing Newton’s remark: “Even the greatest human innovators are, in perspective, midgets standing on the shoulders of a vast pyramid of other midgets.” We would add that the way in which midgets work is not independent from the degree of social coupling among them, and only a rather precise degree of such coupling is consistent with successful discovery by a community of researchers. The efficiency of the search process appears heavily dependent on the weight that agents posit on social influence. Our results suggest that there exists an optimal value for the social coupling parameter that maximizes the ability of the agents to find the superior paradigm as a community. The occurrence of a paradigm shift becomes more likely when each member of the community attaches a small but positive weight to the experience of his/her peers. This way, the conservative force exerted by the representatives of the current paradigm cannot hinder individual discovery and can even empower successful individual pioneers to lead his/her peers towards the superior paradigm.

The ideal scenario to test to a full extent the proposed model would be of course to compare it against empirical data [29]. However, because of the nature of the problem being addressed (how agent interaction affects the rate of adoption of a given technology) such comparison represents an entirely new (empirical) study in itself. In order to perform such comparison while making justice to the question (specifically Fig 3) it would be necessary to have data on the success of a given technology, paradigm, theory, etc. for different levels of interconnectedness among the same scientists (or users). This, however, cannot be obtained in the real world for the same technology and the same users in a common time window. Recent work has addressed a somewhat comparable question by asking how the success of a scientific author depends on his/her position in the co-authorship network [30]. For such work an extensive mining of the author databases, which is beyond the aim of the current work, is necessary. That said, we believe that the results presented in Fig 3 can be interpreted as a prediction of the *degree* of social influence (and not social influence *tout court*) has the better chance of positively influencing the acquisition of a new technology. Specifically, it predicts that when between 10–15% of the influence on each agent’s decisions is attributable to social influence, the *group* will find the best technology in the least time. This prediction should in principle be testable (albeit in a non ideal way) for collaboration networks where the dependent variable is some measure of peer-success of a given product (publication, theory, method, etc.) compared between well-defined, finite sets of authors with different patterns of interaction.

Finally, such results could have policy implications because they refer to the conditions under which knowledge is produced. Although the weight agents attach to the experience of their peers with respect to their own is presented in our model as an attribute of the agents themselves, it could as well be influenced by the environment in which the knowledge community operates. If so, it could be the object of design or intervention by policy makers in order to foster innovation and discovery.

## Supporting Information

S1 File MATLAB Simulation Code File. The MATLAB^®^ (The Mathworks Inc.) code for simulating one migration is provided as supporting information for public use. Both *n* and *α* can be easily modified for comparative dynamics analysis. An animated applet for a fixed group size and different values of *α* has been uploaded at http://www.complejidadsocial.cl/2012/projects/paradigm-shifts/ for readers to visually grasp both micro and macro dynamics of individual paradigm shifts under our framework.
(PDF)
