# Supplementary material for: The Underlying Social Dynamics of Paradigm Shifts
Source: PLoS One. 2015 Sep 29;10(9):e0138172. doi: 10.1371/journal.pone.0138172 (PMC4587884; doi:10.1371/journal.pone.0138172)
Supplement: S1 File — The MATLAB® (The Mathworks Inc.) code for simulating one migration is provided as supporting information for public use. Both n and α can be easily modified for comparative dynamics analysis. An animated applet for a fixed group size and different values of α has been uploaded at http://www.complejidadsocial.cl/2012/projects/paradigm-shifts/ for readers to visually grasp both micro and macro dynamics of individual paradigm shifts under our framework. (PDF) [file pone.0138172.s001.pdf]

```matlab
% The results of the simulation are stored on matrix T.
% T(k,j,r) is the time until shift of realization r, for  n = j*12, and alpha = (j-1)/50.

clear;
clc;

% Parameters of the model:

a = 0.01; b = 2; c = 1; d = 6; e = 200;

epsilon = 0.3665; omega   = pi;

xA = -1.268381098514741;
xB =  0.5265616172625899;
xC =  4.4918194812521515;

xCorte = 4;

% Simulation parameters:

dt = 1/500; % Time differential.
R = 1000;   % Number of realizations.

K = 51;
ALPHA = linspace(0,1,K); % Values for Alpha.
N     = [12 24 36];      % Values for n.

% Simulation:

for r = 1:R % Iterate over realizations.
    for j = 1:3 % Iterate over the values of n
        n = N(j);

        % Initializing matrices:
        G  = zeros(n,1);
        X  = zeros(n,1); % X(i) = position of individual i in the landscape.
        dX = zeros(n,1); % Change in his position.

        for k = 1:K % Iterate over values of alpha.

            alpha = ALPHA(k);
            t = 1;
            X = repmat(xA,[n 1]); % Initially, all individuals are on position xA.

            while 1

                V = a*(-X.*(X+b).*(X-c).*(X-d) + e); % V(i) = height of individual i on the landscape.
                f = -0.04*(-xC + X).*(-xB + X).*(-xA + X); % f(i) = V'(i).

                for i = 1:n
                    G(i)  = sum((V-V(i)).*(X-X(i)))/(n-1);
                    dX(i) = ((1-alpha)*f(i) + alpha*omega*G(i))*dt + epsilon*sqrt(dt)*randn(1);
                end;

                X = X + dX;
                Z = mean(X >= xCorte);

                % Display progress:
                if mod(t*dt,1) == 0
                    disp(sprintf('r = %d  n = %d  alpha = %1.2f  t = %d  Z =%1.2f',r,n,alpha,t*dt,Z));
                end;

                if Z >=0.9
                    break;
                end;
                t = t+1;
            end;
            T(k,j,r) = t*dt;
        end;
    end;
end;
```
